# Straightforward Protein-Protein Interaction Interface Mapping via Random Mutagenesis and Mammalian Protein Protein Interaction Trap (MAPPIT)

**DOI:** 10.3390/ijms20092058

**Published:** 2019-04-26

**Authors:** Laurens Vyncke, Delphine Masschaele, Jan Tavernier, Frank Peelman

**Affiliations:** 1 Cytokine Receptor Laboratory, Flanders Institute of Biotechnology, VIB-UGent Center for Medical Biotechnology, Faculty of Medicine and Health Sciences, Ghent University, 9000 Ghent, Belgium; laurensvyncke@hotmail.com (L.V.); Delphine.Masschaele@uzgent.be (D.M.); jan.tavernier@vib-ugent.be (J.T.); 2 Department of Biomolecular Medicine, Ghent University, B-9000 Ghent, Belgium

**Keywords:** interface mapping, MAPPIT, protein-protein interaction, random mutagenesis

## Abstract

The MAPPIT (mammalian protein protein interaction trap) method allows high-throughput detection of protein interactions by very simple co-transfection of three plasmids in HEK293T cells, followed by a luciferase readout. MAPPIT detects a large percentage of all protein interactions, including those requiring posttranslational modifications and endogenous or exogenous ligands. Here, we present a straightforward method that allows detailed mapping of interaction interfaces via MAPPIT. The method provides insight into the interaction mechanism and reveals how this is affected by disease-associated mutations. By combining error-prone polymerase chain reaction (PCR) for random mutagenesis, 96-well DNA prepping, Sanger sequencing, and MAPPIT via 384-well transfections, we test the effects of a large number of mutations of a selected protein on its protein interactions. The entire screen takes less than three months and interactions with multiple partners can be studied in parallel. The effect of mutations on the MAPPIT readout is mapped on the protein structure, allowing unbiased identification of all putative interaction sites. We have thus far analysed 6 proteins and mapped their interfaces for 16 different interaction partners. Our method is broadly applicable as the required tools are simple and widely available.

## 1. Introduction

**Figure 1:**
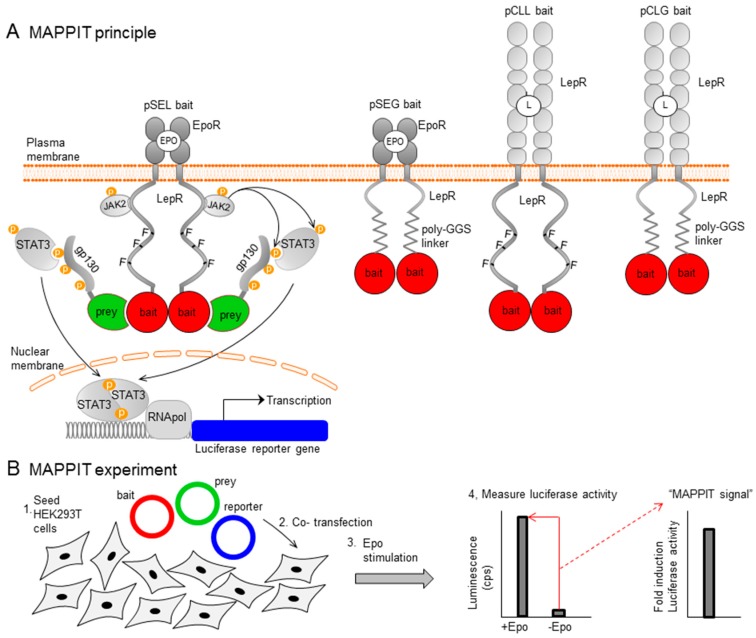
Mammalian Protein Protein Interaction Trap (MAPPIT) (**A**) MAPPIT principle. In the pSEL MAPPIT bait, the bait receptor comprises the extracellular Epo Receptor domain and a mutant intracellular Leptin Receptor domain, lacking STAT3 recruitment sites. For the alternative pSEG and pCLG MAPPIT baits, the intracellular LepR domain C-terminal of the JAK2 binding site is replaced by a glycine-glycine-serine (GGS) linker allowing more flexibility. The pCLL and pCLG baits contain the extracellular LepR domain. (**B**) MAPPIT outline. HEK293T cells are transfected with bait, prey and reporter constructs. 24 h post-transfection, cells are stimulated with cytokine. The next day, luciferase activity (luminescence, in counts per second) is measured. The MAPPIT signal represents the fold induction between stimulated and non-stimulated wells.

The expansion of the protein interactomics field is driven by a wide variety of techniques for the detection of protein-protein interactions (PPIs). Classical techniques, such as yeast two-hybrid [1] and affinity-purification coupled to mass spectrometry [2], have been complemented with new and improved approaches. These enable the study of PPIs at their subcellular location [3,4] or in a physiological context between weak and transient interactions [5,6,7], full-length integral membrane proteins [8] and post-translationally modified proteins [9]. Our laboratory developed the mammalian protein-protein interaction trap (MAPPIT), a method combining many of these traits. MAPPIT is based on the Janus kinases-signal transducers and activators of the transcription (JAK-STAT) signalling pathway of type I cytokine receptors upon bait and prey interaction (Figure 1A). The MAPPIT bait is fused to a signalling-deficient chimeric receptor comprising an extracellular cytokine receptor domain and a mutant intracellular receptor domain, lacking STAT3 recruitment sites. The prey is coupled to a gp130 fragment. Upon cytokine stimulation, bait-prey interaction leads to phosphorylation and the recruitment of STAT3 to the gp130 fragment, complementing the JAK-STAT signalling pathway, resulting in luciferase reporter gene activation. As MAPPIT acts in intact mammalian cells, it allows the detection of proteins requiring intracellular cofactors or post-translational modifications (PTMs) [10]. A typical MAPPIT experiment simply involves co-transfection of three plasmids in HEK293T cells: a bait, a prey, and a STAT3-inducible luciferase reporter plasmid (Figure 1B). Interaction between bait and prey protein is detected by luciferase activity induced after stimulation with erythropoietin (Epo) or leptin, depending on the extracellular domain of the bait. MAPPIT can detect up to one third of all protein interactions in high-throughput screening modus but this number can probably be increased by switching bait and prey, using different bait constructs or using domains instead of full-length proteins [11,12,13]. 

High-resolution structure determination of protein complexes via X-ray crystallography, nuclear magnetic resonance (NMR) spectroscopy or cryo-electron microscopy is inherently slow and is often very challenging. Even with major advances and speedup in structure determination, these methods cannot keep up with the rapid growth of the interactome [14]. This leads to a huge and growing gap between the number of known protein interactions and the number of structurally characterized complexes. 

Alternatively, many approaches can predict protein complex structures based on the separate models or structures of the interacting proteins in the complex [14,15]. Several methods provide experimental insight into the location of the protein interaction interface [15]. Examples include nuclear magnetic resonance (NMR) titration/chemical shift perturbation experiments or mass spectrometry combined with limited proteolysis, chemical crosslinking or deuterium exchange. Similarly, mutagenesis combined with a detection method for protein interactions can identify critical interacting residues. Combining experimental insights into the location of the interacting interfaces with homology modelling or in silico protein-protein docking, enables the building of reliable models for the protein complex [15,16]. 

In this paper, we describe a straightforward method combining random mutagenesis with MAPPIT that allows extensive mapping of PPI interfaces in intact human cells. 

## 2. Materials and Methods

**Figure 2:**
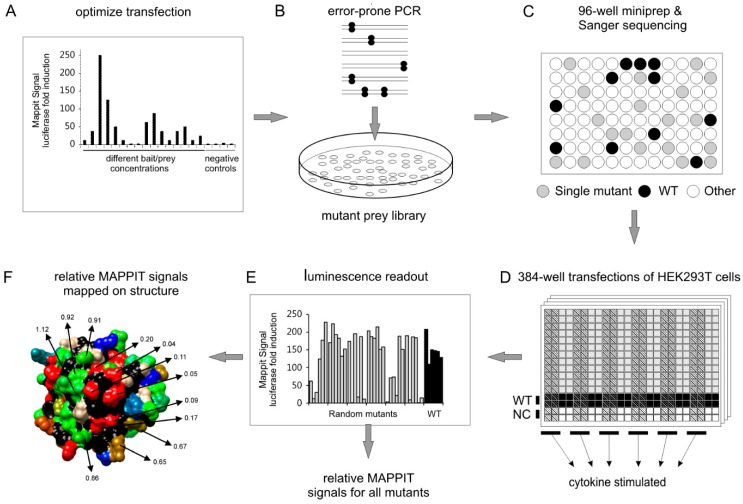
6-step workflow of the MAPPIT-based interface mapping method. (**A**) MAPPIT transfection conditions are optimized by varying the concentration of bait and prey plasmids and by switching the bait and prey proteins. (**B**) The target protein is randomly mutated via error-prone polymerase chain reaction (PCR). The linear PCR product is cloned into the MAPPIT prey plasmid vector and electroporated into *E. coli* DH10B cells, resulting in the MAPPIT mutant prey library. (**C**) Individual *E. coli* colonies are inoculated in 96-deepwell blocks. Mutant prey DNA is isolated via a 96-well DNA miniprep protocol and sequenced by Sanger sequencing. (**D**) MAPPIT prey mutants harbouring a single missense mutation are co-transfected with the MAPPIT bait and STAT3 luciferase reporter plasmids in 384-well plates seeded with HEK293T cells. (**E**) After cytokine stimulation via Epo or leptin, the luciferase activity is measured and relative MAPPIT signals are calculated. (**F**) The relative MAPPIT signal of each mutant is determined and mapped on the protein structure.

Figure 2. summarizes the 6-step workflow of our method. Below, we describe the six steps in more detail.

### 2.1. Step 1: Optimization of the MAPPIT Readout

We first set up and optimize MAPPIT assays that specifically detect the interaction between the protein of interest and its interaction partners (Figure 2A). Their cDNA is cloned in both MAPPIT bait and MAPPIT prey plasmid vectors. The MAPPIT assay is optimized by testing the protein of interest both as bait and as prey and by varying the concentration of bait and prey plasmids. To ensure the specificity of the assay, baits and preys are also tested versus negative control baits and preys, expressing an irrelevant protein. The induction of luciferase activity upon cytokine stimulation should be at least 10-fold. Both prey and bait can be randomly mutated. Below, we describe the screening process, in which the mutation target is cloned as prey. 

### 2.2. Step 2: Random Mutagenesis via Error-Prone PCR

The DNA insert is randomly mutated via error-prone PCR using Mutazyme II DNA polymerase, following the guidelines of the Genemorph™ II Random Mutagenesis kit (Agilent Technologies, Santa Clara, CA, USA) (Figure 2B). This permits a suitable mutation rate and a balanced mutation spectrum [17]. A good balance between all possible types of mutations (e.g., A to T vs G to C etc.) is important to ensure that every codon has a good probability of being mutated and to ensure that every type of mutation is allowed. This allows good coverage of different types of mutations spread over the entire protein. To obtain a maximum number of single missense mutants, the PCR condition is first optimized by varying the concentration of input DNA and the number of PCR cycles. The PCR primers contain unique restriction sites allowing in-frame ligation of the linear PCR product into the MAPPIT prey plasmid vector. The ligation product is electroporated into *E. coli* DH10B cells. For each PCR condition, 24 colonies are grown overnight in a 2× Yeast Tryptone medium in a 96-deepwell block. DNA is purified via a 96-well miniprep protocol using the Nucleospin™ Robot-96 plasmid kit (Machery Nagel, Easton, PA, USA). Next, the MAPPIT mutants are sequenced on Applied Biosystems 3730XL DNA Analyzers to determine the PCR condition with the highest number of single missense mutations. In our studies, up to one third of the random mutant clones contain single missense mutations [18,19].

### 2.3. Step 3: Generation of a Mutant Plasmid Library in 96-Well Format

Mutant *E. coli* colonies of the optimal PCR condition are plated and single colonies are inoculated in 96-deepwell blocks for DNA miniprep purification, as described above (Figure 2C). In the automated DNA minipreps, DNA is eluted in water into UV-transparent flat-bottom 96-well plates and DNA concentration is measured via the Magellan UV spectrophotometer (Tecan, Männedorf, Switzerland). The OD 260/280 ratio should be above 1.8 for every sample to obtain reliable MAPPIT results. Next, DNA is normalized to the optimized concentration for transfection. The sequences of all randomly mutated plasmids are determined via Sanger sequencing and plasmids with a single missense mutation are transferred into 96-well plates (Figure 2C). One row of each 96-well plate contains plasmids with a wildtype prey and one row contains a negative control prey (Figure 2D).

### 2.4. Step 4: MAPPIT in 384-Well Assay

The normalized 96-well MAPPIT prey plates are co-transfected with the MAPPIT bait and STAT3 luciferase (pXP2d2-rPAPI-luciferase) reporter plasmids, as optimized in step 1 (Figure 2D). On the first day, 3000 HEK293T cells/well are seeded into black 384-well plates. The next day, cells are transfected with the MAPPIT prey together with a mixture of MAPPIT bait and STAT3 luciferase reporter plasmids using a calcium phosphate precipitation method. Each unique prey/bait mixture is transfected in 8 384-wells. One 96-well plate with prey mutant DNA thus requires transfection of two 384-well plates. As the optimized 96-well DNA plates contain 12 wildtype and 12 negative control prey plasmids, each transfected 384-well plate contains 6 different wildtype preys and up to 36 different prey mutants. 

On day three, 4 out of 8 wells for each transfection mixture are stimulated with leptin or Epo. The 4 other wells are left unstimulated (Figure 2D). After 24 h, cells are lysed in 15 µL of Cell Culture Lysis Reagent buffer (25 mM Tris/phosphate (pH 7.8), 2 mM DTT, 2 mM CDTA, 10% glycerol, 1% Triton X-100) followed by the addition of 11 µL of luciferase substrate buffer (40 mM Tricine, 2.14 mM (MgCO_3_)_4_Mg(OH)_2_·5H2O, 5.34 mM MgSO_4_·7H_2_O, 66.6 mM DTT, 0.2 mM EDTA, 270 µM coenzyme A (Sigma-Aldrich, St. Louis, MO, USA), 530 µM ATP (Sigma-Aldrich, St. Louis, MO, USA), 470 µM luciferin (Duchefa, Haarlem, The Netherlands). The luminescence is measured with the EnSpire plate reader (PerkinElmer Life Sciences, Waltham, MA, USA).

### 2.5. Step 5: Data Analysis

First, the “MAPPIT signal” is determined by dividing the average of the luciferase counts of the 4 stimulated wells by the average of the luciferase counts of the 4 unstimulated wells (Figure 2E). Then, the “normalized MAPPIT signal” of each mutant is calculated by dividing its MAPPIT signal by the median of the MAPPIT signals of the 6 wildtype preys on the same 384-well plate. As each MAPPIT transfection experiment is performed three times, the “relative MAPPIT signal” of each mutant is determined by the average of the normalized MAPPIT signals in these three experiments.

**Figure 3:**
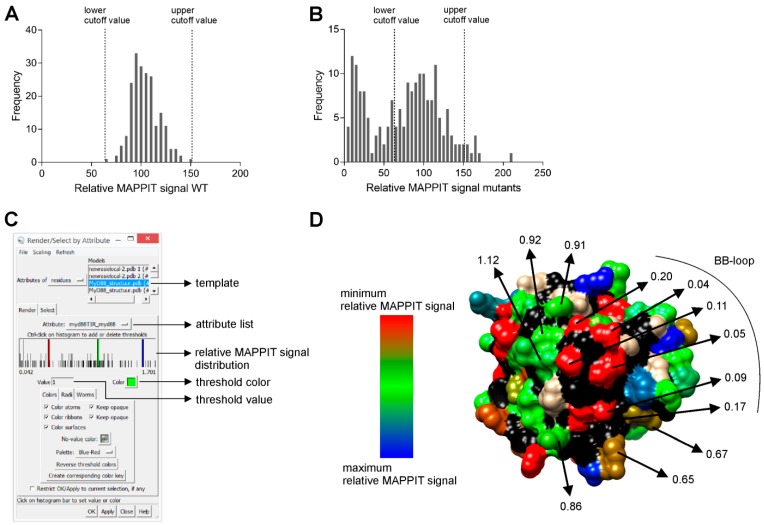
Random mutagenesis of the MyD88 TIR prey–MyD88 bait interaction. (**A**) Relative MAPPIT signal distribution of all MyD88 TIR wildtypes (WTs) for MyD88 TIR prey-MyD88 bait interaction. (**B**) Relative MAPPIT signal distribution of all unique single mutants for MyD88 TIR prey-MyD88 bait interaction. (**C**) The “render by attribute” tool of Chimera. (**D**) Effect of mutations on the MyD88 TIR prey-MyD88 bait interaction. Residues are coloured on the MyD88 TIR crystal structure according to their relative MAPPIT signal [21]. A cluster of red-coloured residues forms a potential binding site (BB-loop). Green-coloured residues do not alter the interaction compared to the wildtype. Non-mutated residues are grey and backbone atoms are black.

We also calculate the relative MAPPIT signals of the wildtypes, as described above, and plot the variation. The lowest and highest wildtype relative MAPPIT signal determines lower and upper cut-off value, respectively. (Figure 3A). The MAPPIT signal of each wildtype is divided by the median of the MAPPIT signals of the 5 other wildtypes on the same 384-well plate. Mutants with a relative MAPPIT signal below the lower cut-off value or above the upper cut-off value are considered to have an effect on the interaction (Figure 3B). 

### 2.6. Step 6: Mapping of Relative MAPPIT Signals

First, we create an “attribute list” in a fixed .txt format in which the relative MAPPIT signal of a mutant is assigned to its corresponding residue number. This attribute list is then loaded in the free modelling program University of California San Francisco (UCSF) Chimera via its “define attribute” tool [20]. The relative MAPPIT signals of the mutated residues are mapped on the template structure using the “render by attribute” tool (Figure 3C). Based on the histogram of the relative MAPPIT signals, up to three threshold values with respective colours can be determined. The colour intensity of each mutated residue depends on the proximity of its relative MAPPIT signal to the thresholds in the histogram. 

## 3. Results and Discussion

### 3.1. MAPPIT Detects Interfaces in Diverse Target Proteins 

We successfully applied random mutagenesis combined with MAPPIT for the interaction interface analysis of six targets in four unrelated protein families: the antiviral host restriction factor apolipoprotein B messenger RNA-editing catalytic polypeptide-like G (Apobec3G) [22], the ring finger protein 41 (RNF41) [23], the ligand binding domain of peroxisome proliferator-activated receptor α (PPAR-α) and the Toll/IL-1R (TIR) domains of the Toll-like receptor adapters MyD88 adapter-like (Mal) [18], myeloid differentiation primary response gene 88 (MyD88) [19] and TIR-domain-containing adapter inducing interferon-β (TRIF). For five of these targets, we tested the interaction of the mutant libraries with three or four different interactors in parallel. We thus mapped interaction interfaces for 16 protein-protein interactions. Figure 3 shows an example of a MAPPIT random mutagenesis screen, testing the interaction of MyD88 coupled as bait with random mutants of the prey MyD88 TIR domain. A mutant library of 17 96-well plates led to 185 unique single missense mutants, resulting in a coverage of 78% of the entire MyD88 TIR domain. No MyD88 TIR wildtype has a relative MAPPIT signal below 60% or above 155%, which were used as lower and upper cut-off values, respectively (Figure 3A). Figure 3B illustrates that 37% of all unique single missense mutants has a relative MAPPIT signal below the lower cut-off value. Figure 3D shows one of the 4 protein interaction interfaces detected in this screen. Residues in the centre of this interface have a relative MAPPIT signal well below the 60% cut-off. Typical for all interfaces found in the screens, mutations at the edge of the interface often have a relative MAPPIT signal between the lower cut-off value and the average wildtype relative MAPPIT signal. To confirm these effects, we re-isolate DNA of the putative interface mutations and new wildtype clones, and retest these selected samples in triplicate MAPPIT experiments.

### 3.2. Interfaces Detected via MAPPIT Are Confirmed via Other Methods and Studies

All tested mutants are available as individual clones and are easily cloned into other vectors. This permits retesting of a large number of mutations in different assays without the need of extra mutagenesis. The biological importance of new potential interfaces identified via MAPPIT was confirmed via orthogonal assays, such as co-immunoprecipitation and signalling assays [18,19]. Protein interfaces identified via our method are well in line with other studies. We confirmed the well-known importance of the TIR “BB-loop” in TIR interactions of MyD88, Mal, and TRIF (Figure 3D) [18,19]. In agreement with other studies, MAPPIT analysis demonstrated that one of two crystallographic interfaces in the Mal TIR crystal structures is a Mal homodimerization interface [18]. By mapping our MAPPIT data on a homology model for Apobec3G, we identified an extensive Apobec3G homodimerization site, as found in parallel studies [22]. We demonstrated that this complete area is also critical for the interaction with the viral infectivity factor (Vif) of HIV, later confirmed by the NMR structure of the Apobec3G N-terminal domain [24].

### 3.3. MAPPIT and Mutagenesis Provide Insight in Disease Mechanisms

**Figure 4:**
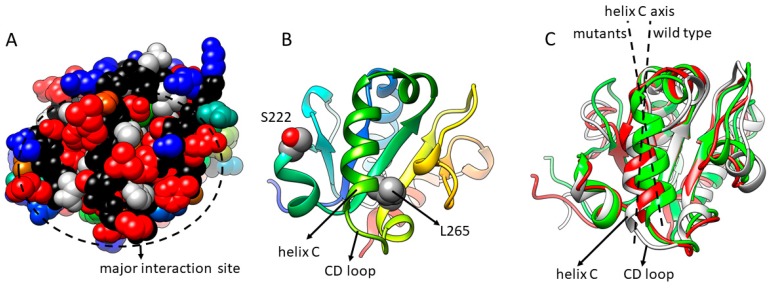
Mutations in two very different diseases both affect MyD88 interactions by affecting the same interaction surface. (**A**,**B**) Random mutations that affect the interactions of MyD88 (red) cluster around the C-terminal half of helix C and the CD loop. Panel A shows a space filling model of the MyD88 TIR domain, with a major interaction site at helix C and the CD loop oriented towards the viewer. Mutations that strongly disrupt interactions with the adapter protein Mal are red. Panel B shows a ribbon model in the same orientation, with indication of helix C, the CD loop, S222 and L265. (**C**) In the L265P mutant (red) and S222R mutant (green), the axis of helix C has tilted, and the CD loop shifted when compared with the wild type (grey ribbon).

Our MAPPIT studies could nicely reproduce effects that were observed in vivo. As a viral defence against the human host restriction factor Apobec3G, this protein is degraded by HIV-1 Vif via ubiquitination mechanisms. A D128K mutation protects simian Apobec3G against degradation via HIV-1 Vif. Via MAPPIT, we could demonstrate that the Apobec3G D128K mutation specifically breaks the Apobec3G-Vif interaction [25]. Mice containing mutations in the TIR domain of TLR4 have a reduced TLR4 innate immune response and are susceptible to infections [26]. Via MAPPIT, we could confirm that these mutations disrupt the TIR-TIR interactions of the TLR4 TIR domain [18]. This suggested that MAPPIT can be used to help to explain the underlying cause of phenotypes in diseases. Our study of MyD88 illustrates best how MAPPIT combined with random mutagenesis indeed helps to understand the molecular mechanism of disease (Figure 4). A somatic L265P mutation in MyD88 is found in more than 90% of all Waldenström macroglobulinaemia B-cell malignancy patients [27]. Using MAPPIT, we demonstrated that the L265P mutation and a S222R mutation both lead to increased interactions between the MyD88 TIR domains, which appear to be associated with increased NF-κB activation [19]. The S222R mutation was generated by random mutagenesis but was almost simultaneously found as a de novo mutation in a unique case of severe juvenile arthritis [28]. Via molecular dynamics, we could demonstrate that both mutations induce a significant tilt of the alpha C-helix and rearrangement of the CD loop [19,28]. Random mutagenesis and MAPPIT analysis of MyD88 demonstrated that helix C and part of the CD loop are central in one of the three major interaction surfaces of the MyD88 TIR domain (Figure 4A,B) [19]. This suggests that two mutations in two very different diseases induce the hyperactivation of MyD88 by modifying one of its interaction surfaces, priming it for interaction.

Mutations can affect an interaction by an effect on folding or stability. For important interpretations of mutants in diseases, such folding/stability effects should be tested experimentally. Mutations can also affect an interface via local folding effects, which may be harder to detect. For example, the L265P mutation was predicted to destabilize the protein by FoldX predictions [19,29]. Molecular dynamics simulations suggested that the mutation indeed causes a local structural change, which in this case probably enhances the MyD88-MyD88 interactions.

While MAPPIT can help to demonstrate defects in protein interactions by disease-associated mutations, proper interpretation of disease-causing mutations requires additional studies, such as orthogonal interaction assays, and determination of expression level and turnover, activity assays and if possible in vivo studies.

### 3.4. Interactions Requiring Additional Ligands or Phosphorylation Can Be Studied via MAPPIT

MAPPIT operates in intact mammalian cells, providing a natural environment with endogenous cofactors and regulatory proteins, and is therefore able to detect protein interactions requiring PTMs such as phosphorylation. Several observations indicate that MAPPIT baits and preys behave like their unmodified proteins, and that they can be the target of normal post-translational modifications like phosphorylation and ubiquitination. The Vif prey is rapidly degraded like untagged Vif, which is a target of proteasomal degradation via ubiquitination. Using Vif as MAPPIT prey required introduction of a mutation in the Vif SOCS box, abrogating its function in an E3 ubiquitin ligase complex, and preventing degradation of the Vif prey [25]. A MyD88 MAPPIT bait induces a very strong NF-kB activation, indicating that MyD88 contains its normal signalling capacity as a bait, and that a signalling pathway that includes extensive phosphorylation and poly-ubiquitination can emanate from the bait. Via inhibitory and phosphomimetic mutations of phosphorylated residues in the MAPPIT bait or prey, we were able to study the crucial role of phosphorylation on protein interactions [19,22]. MAPPIT also allowed to study interactions of Apobec3G that required cellular RNA as additional ligand [22], while interactions of PPAR-α could only be studied after adding an exogenous ligand. Of note, in cases in which interactions require a modification that does not naturally occur by overexpression of bait or prey, the modifying enzyme can be co-expressed as part of an extra receptor in heteromeric MAPPIT, as demonstrated for the serine-phosphorylation dependent interactions of SMAD proteins in the TGF-β family [30].

### 3.5. Critical Considerations in Data Interpretation

The MAPPIT signal is also affected by mutations disrupting the structure or proper folding of the protein. For example, most mutations in the core of the protein affect the MAPPIT signal. As mutations at the protein surface can equally disrupt the protein structure, it is mandatory to carefully inspect possible structural effects. As a tool for estimating the mutational effect on the protein structure, we use the FoldX prediction program [29]. However, in silico predictions remain quite unreliable. Users should consider that the effects of very small clusters of mutations can be the result of altered stability or folding, even if FoldX does not predict such effects. Moreover, errors in published structures cannot be excluded, which may make structural interpretation and correct FoldX estimation more difficult. In this context, any mutation of an isolated surface residue or very small patch of residues should be considered with caution. Potential interface areas rather present themselves as patches of multiple adjacent residues with missense mutations that all affect the interaction (Figure 3D). 

We analyse expression levels of prey or bait mutants to exclude an effect on the MAPPIT interaction due to differential expression. Expression levels of MAPPIT preys can be determined via Western blot, as these contain a Flag-tag. Expression of the MAPPIT baits can be determined in a (parallel) MAPPIT experiment with a MAPPIT prey specifically recognizing the receptor portion or JAK2 portion of the bait. However, expression levels of bait or prey mutants are not a good measure for their proper folding or structural integrity. Very often, mutations that clearly disrupt the structure or folding of the protein hardly affect the expression level.

Our method identifies all regions required for protein interaction and some of these do not necessarily represent the actual interface between bait and prey. This is relevant for proteins binding their target as a homodimer or requiring a third interaction partner. In MAPPIT, both bait and prey proteins are free to homodimerize or even homo-oligomerize. We do find that mutations in the homodimerization interface of the mutated target affect its heterotypical interaction. Similarly, for interactions that require an extra third protein or other ligand, mutations in the binding site of these ligands also affect the MAPPIT readout [19]. 

In this respect, NMR methods, crystallography and cryo-EM can reveal the details of the actual interface between proteins, while extensive mutagenesis can only indicate the position of the interfaces on the interaction partners, without revealing how these surface areas interact to form the interface. However, our method does reveal how specific mutations affect the interaction, which cannot be directly deduced from most other methods and can be very complementary with structure determination.

## 4. Concluding Remarks

Our combination of MAPPIT and random mutagenesis allows the extensive and unbiased validation of interfaces in high- or low-resolution structures. Interface residues identified via MAPPIT can also guide the docking process in in silico data-driven protein-protein docking [19]. The method helps to understand the mechanism of disease-linked mutations. A major advantage of our screening method lies in its simplicity and rapid setup. The entire optimization and screening process can be performed in three months. We therefore believe that our MAPPIT-based interface mapping method is an excellent tool for the detailed study of a large number of PPIs.

## Abbreviations

Apobec3G	apolipoprotein B messenger RNA-editing catalytic polypeptide-like G
Epo	erythropoietin
gp130	glycoprotein 130
JAK	Janus kinase
LepR	leptin receptor
Mal	MyD88 adapter-like
MAPPIT	mammalian protein-protein interaction trap
MyD88	myeloid differentiation primary response gene 88
NMR	nuclear magnetic resonance
PPAR-α	peroxisome proliferator-activated receptor α
PPI	protein-protein interaction
PTM	post-translational modification
RNF41	ring finger protein 41
STAT	signal transducers and activators of transcription
TIR	Toll/interleukin-1 receptor
TRIF	TIR-domain-containing adapter inducing interferon-β
